# Bilateral Pulmonary Artery Mycotic Aneurysms in a Female with Infective Endocarditis

**DOI:** 10.7759/cureus.5114

**Published:** 2019-07-10

**Authors:** Kumail Khandwala, Hunaina Shahab, Dawar B Khan, Saira Bokhari

**Affiliations:** 1 Radiology, Aga Khan University Hospital, Karachi, PAK; 2 Cardiology, Aga Khan University Hospital, Karachi, PAK

**Keywords:** infective endocarditis, mycotic aneurysm, echocardiography, computed tomography

## Abstract

We present a case of a young female who had large bilateral pulmonary artery aneurysms and was found to have infective endocarditis on echocardiography. Endovascular treatment was sought; however, was not possible due to severe sepsis and other associated complications. Subsequent serial imaging revealed the rapid enlargement of one of the aneurysms and possible rupture which proved to be fatal. Although pulmonary artery aneurysms are a rare vascular anomaly, they are seen in a wide variety of conditions, such as congenital heart disease, infection, trauma, pulmonary hypertension, cystic medial necrosis, and generalized vasculitis. This case highlights the consequences of mycotic aneurysms and emphasizes the importance of prompt diagnosis. Early angioembolization and/or surgical repair is often essential to avoid death from rupture of the aneurysm.

## Introduction

The term “mycotic aneurysm” was first described in 1885 and has been used to describe any infectious process leading to aneurysmal dilatation of the arterial wall [1]. Although pulmonary artery aneurysms are a rare vascular anomaly, they are seen in a variety of conditions, such as congenital heart disease, infection, trauma, pulmonary hypertension, cystic medial necrosis, and vasculitis. When present, these lesions tend to grow rapidly with the risk of rupture and death [2]. Surgical or endovascular treatment is recommended whenever possible although the spontaneous resolution has also been reported occasionally for smaller aneurysms [3-4]. We report a case of a young female with bilateral pulmonary aneurysms, initially thought to be attributed to vasculitis, such as that associated with Behçet’s disease; but mycotic pulmonary artery aneurysms were later established as the diagnosis after identification of valve vegetation on echocardiography. We aim to review the literature on the disease etiology, radiological findings, and management options.

## Case presentation

**Figure 1:**
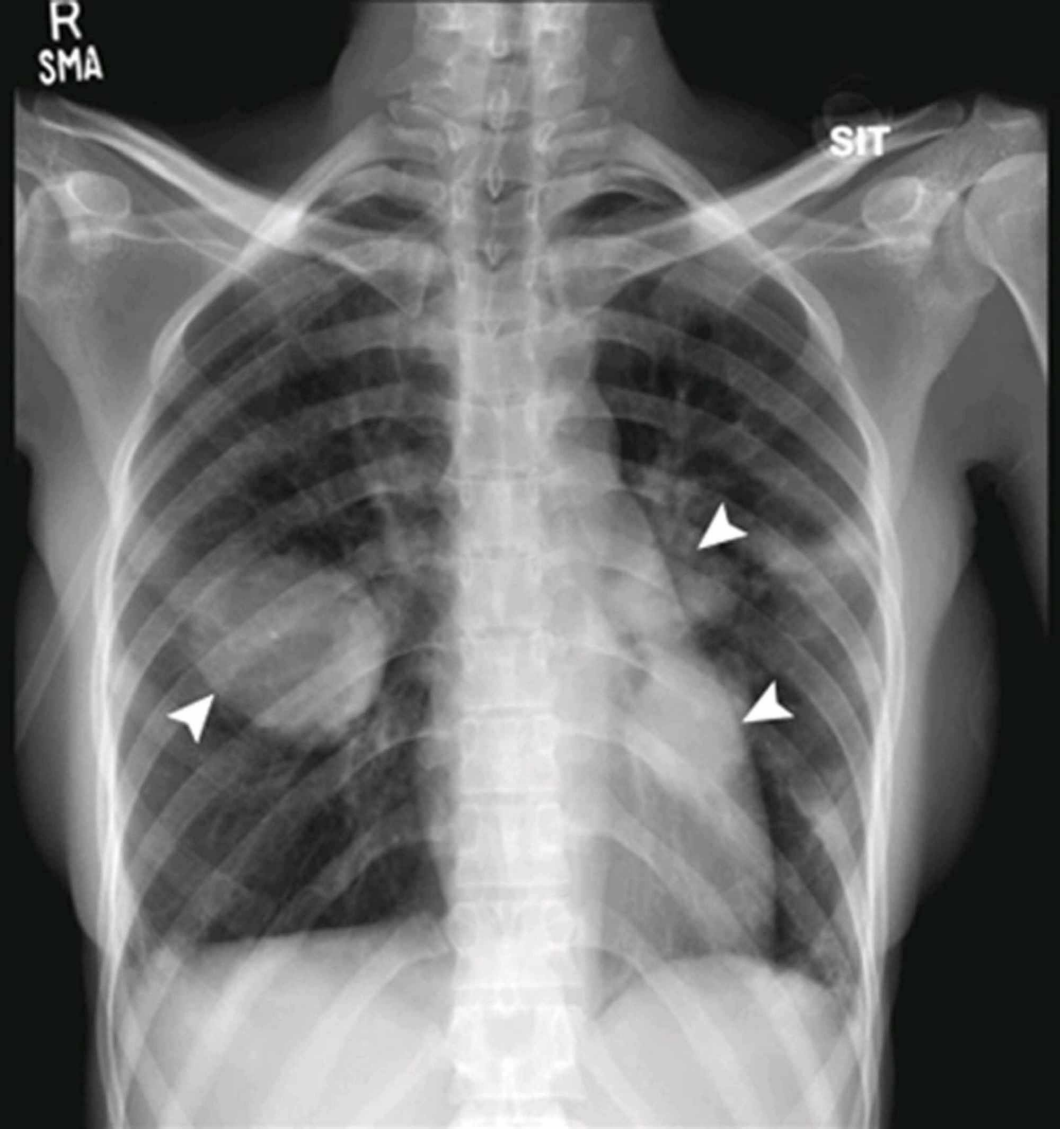
Bilateral perihilar rounded opacities were noted on initial chest x-ray (arrowheads).

A 25-year-old female, known to be a water-pipe smoker, presented to the Aga Khan University Hospital, Emergency Room (ER) with the complaints of low-grade fever for the past two months along with shortness of breath and dry cough for the past two weeks. She had lost around three kilograms of weight over the past two months. She denied any history of tuberculous contact, joint pains, rash, or oral ulcers. She was single with no prior sexual history and denied drug abuse. Based on her history, she had been started on empiric anti-tuberculous therapy at another hospital which she took for 15 days before presenting to our hospital due to lack of symptom resolution. Her initial workup included a chest x-ray which showed large bilateral rounded perihilar opacities (Figure 1).

On presentation, she was found to have a pulse of 120 beats per minute, blood pressure of 80/60 mm Hg, respiratory rate of 30 per minute, the temperature of 39 degrees Celsius and she could maintain a saturation of 96% on five liters oxygen via face-mask. Her general physical examination was significant for pallor, dehydration, a tattoo on the left forearm along with some suspicious needle marks in the same location. On chest auscultation, she was found to have bilateral harsh vesicular breathing. Her cardiac examination was unremarkable, with a normal S1 and S2 and no added sounds or murmurs. The abdomen was soft, non-tender with no visceromegaly.

**Figure 2:**
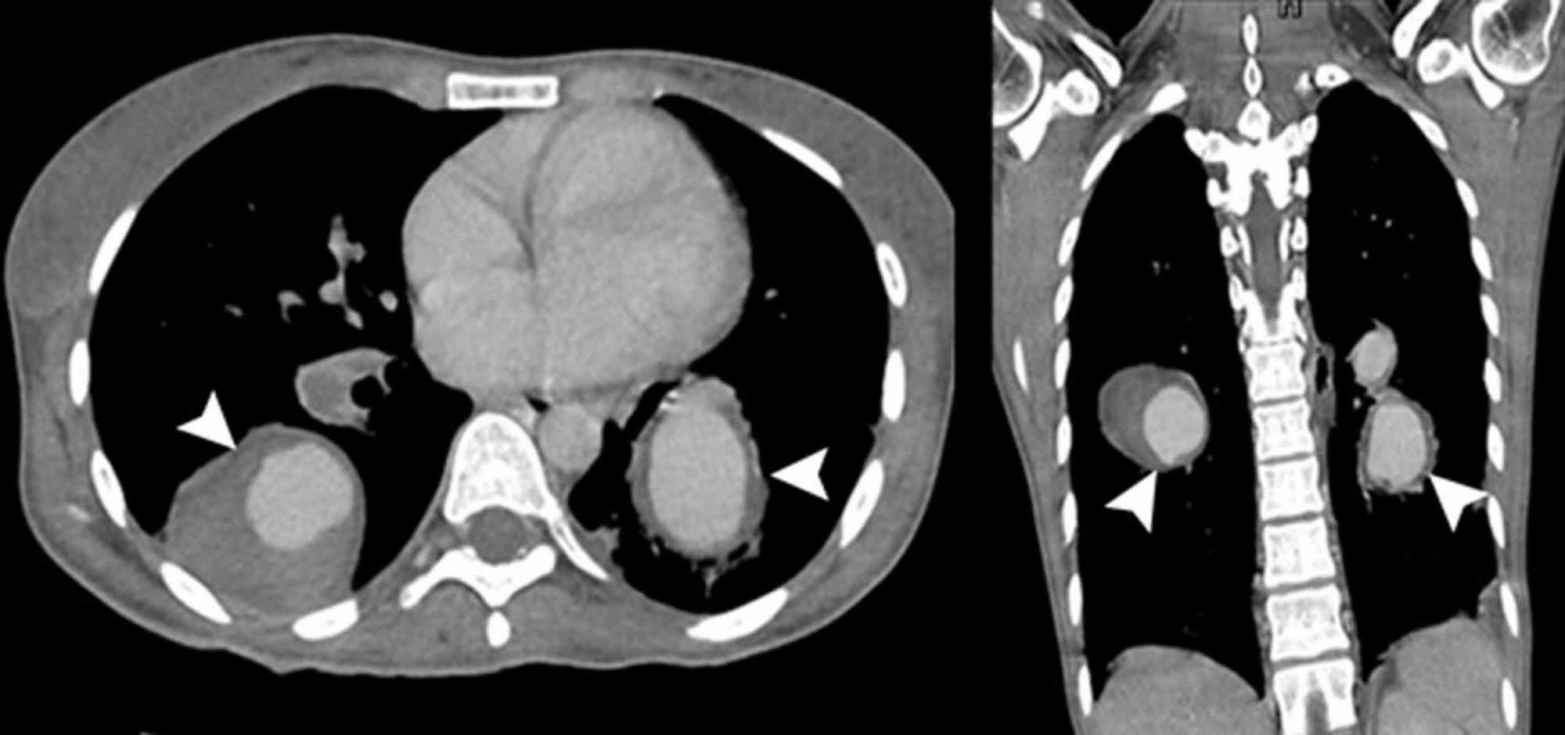
Computed tomography images showing large bilateral partially thrombosed aneurysms (arrowheads) originating from the pulmonary arteries.

She was immediately admitted to a special care unit and started on intravenous (IV) fluids and IV antibiotics after blood workup including cultures were sent. A central venous pressure (CVP) line was passed and she was started on IV norepinephrine. Her blood workup revealed hemoglobin of 8.5 g/dL (normal: 12-15.5 g/dL), white cell count of 27.6 x 10E^9 ^(normal: 4.5-11.0 x 10^9^), and a normal platelet count. Urine analysis was normal. Her procalcitonin was positive at 3.57 ng/mL (normal: <0.15ng/mL) and C-reactive protein (CRP) was 26.7 mg/dL (normal: <3.0mg/dL). Rheumatology workup including anti-nuclear antibodies (ANA), anti-double stranded DNA (anti-dsDNA), anti-smooth muscle antibodies (ASMA), anti-mitochondrial antibodies (AMA), perinuclear anti-neutrophil cytoplasmic antibodies (p-ANCA), cytoplasmic antineutrophil cytoplasmic antibodies (c-ANCA), and anti-cardiolipin immunoglobulin M and immunoglobulin G were all negative. Human immunodeficiency virus (HIV) 1 and HIV 2 assays were negative. Brucella abortus and Brucella melitensis titers were <1:80. Echinococcus immunoglobulin G assay was negative. A computed tomography (CT) scan chest with contrast was done which showed bilateral pulmonary arterial aneurysms (Figure 2).

The aneurysm on the right side was saccular and originating from the inferior division of the right pulmonary artery. It was measuring 5 x 4 cm with peripheral thrombosis. Two aneurysms were identified on the left side. The saccular aneurysm originating from the left main pulmonary artery was measuring 3 x 2.8 cm, lying just adjacent to the left main bronchus. This aneurysm was also showing peripheral thrombosis. Another large saccular aneurysm was originating from the inferior division of the left pulmonary artery which was abutting the left lower lobe bronchus and was measuring 3.5 x 3.7 cm. A subpleural wedge-shaped opacity was identified within the left upper lobe and the right middle lobe. Otherwise, the lung fields did not show signs of consolidation, infiltrates, or cavitation. On radiological findings, a differential of aneurysmal dilatation secondary to vasculitis like Behçet Syndrome was given, however, the laboratory workup and clinical findings did not support this diagnosis.

**Figure 3:**
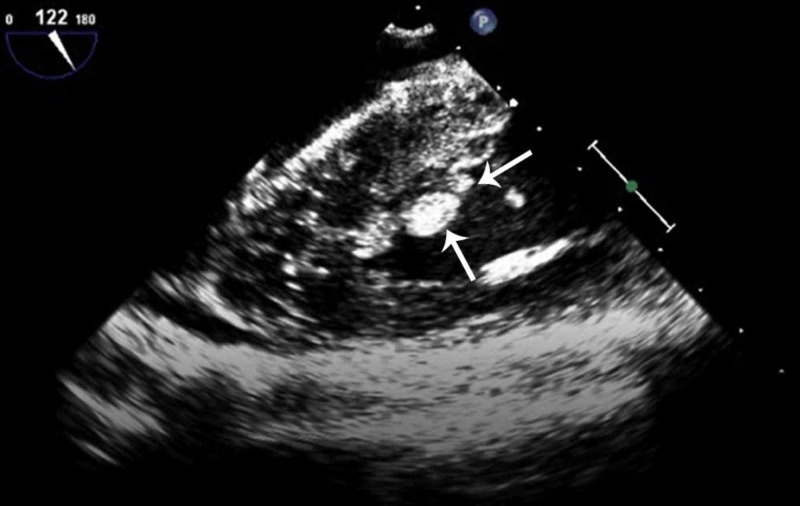
Transesophageal echocardiogram image showing the right ventricle and the tricuspid valve. Two of the four echogenic densities/ masses attached to the chordae are also seen (white arrows).

The patient was transfused one packed red cells and was started on therapeutic enoxaparin for partial thrombosis in the pulmonary arteries. Non-invasive ventilation was initiated. Transthoracic echocardiogram was done which showed a normal ejection fraction of 55%, mild pulmonary artery hypertension, and mild tricuspid regurgitation. A small echogenic density was noted on the tricuspid valve suggestive of vegetation. In order to confirm infective endocarditis, a transesophageal echo was done which showed small vegetation on the posterior leaflet of the tricuspid valve and four large echogenic densities attached to the chordae of the right ventricle (Figure 3). Due to infective endocarditis, therapeutic anticoagulation was discontinued. 

The patient continued to spike fever and CRP continued to increase. IV antibiotic coverage was broadened and IV antifungals were added. She suddenly developed massive hemoptysis along with blood clots followed by pulseless electrical activity. Cardiopulmonary resuscitation (CPR) was conducted for eight minutes during which she was transfused blood and intubated after which she achieved the return of spontaneous circulation. She remained comatose and developed jerky movements of the body. Magnetic resonance imaging of the brain was done which showed hypoxic brain injury.

**Figure 4:**
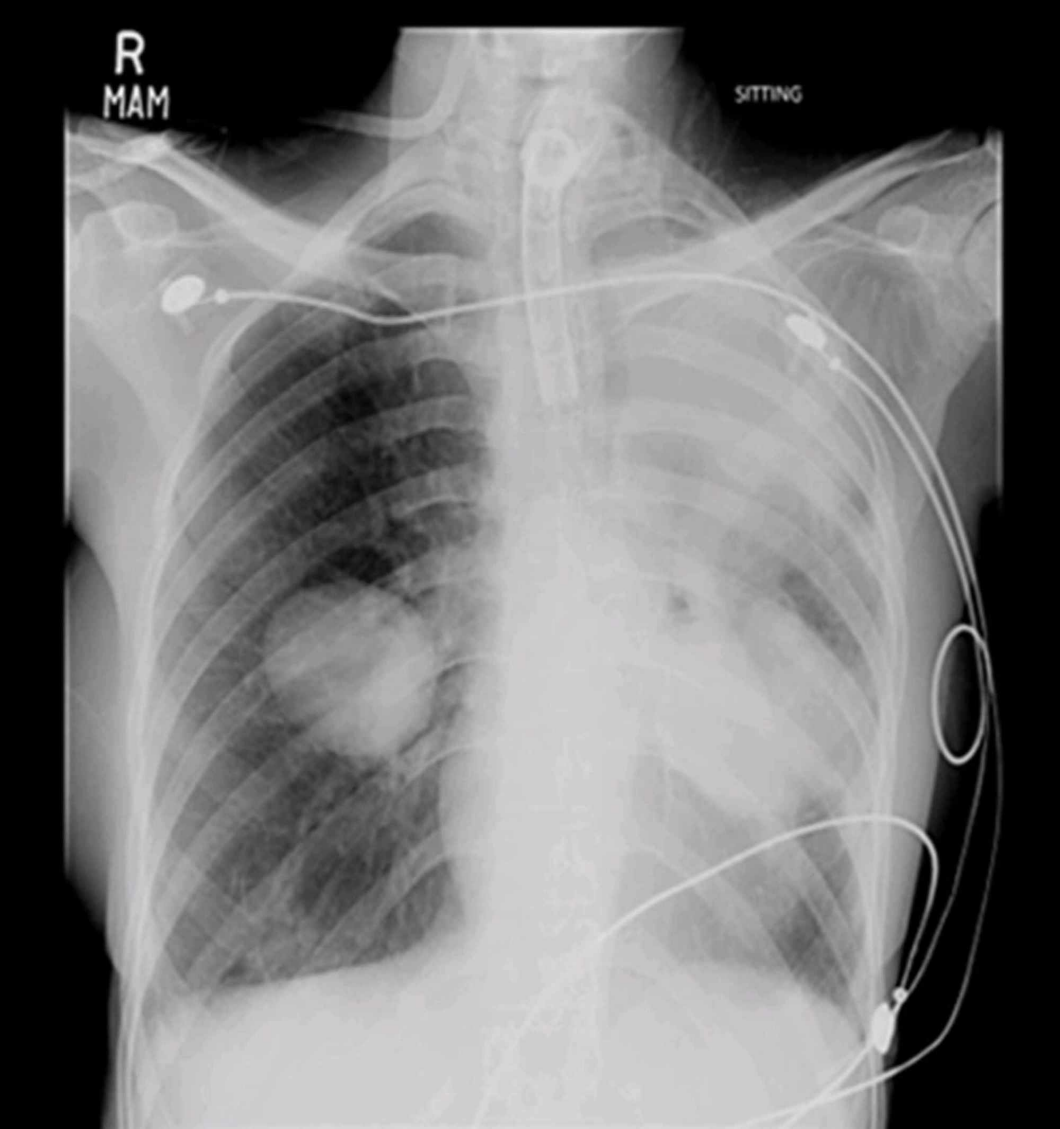
Whiteout identified in the left hemithorax with enlargement of the aneurysms, likely representing hemorrhage secondary to rupture.

She failed extubation trial and was switched to chronic ventilation after performing a tracheostomy. She kept bleeding from the tracheostomy tube due to further pulmonary artery aneurysm rupture and blood was transfused accordingly. In view of guarded prognosis, her code was changed to do not resuscitate (DNR) after counseling and consent of the family. Surgical or endovascular treatment was not sought further due to these existing complications. Her blood cultures failed to reveal any organism growth. Her chest x-ray showed an increase in the size of opacities with haziness in the left lung suggesting rupture of the aneurysm (Figure 4). She then developed pulseless electrical activity and asystole. CPR was not done due to the code status, after which death was declared to the family after three days of admission.

## Discussion

Mycotic pulmonary artery aneurysm is a rare complication of right-sided endocarditis. Most of the cases in the literature are associated with endocarditis due to congenital heart diseases or intravenous drug addiction [2-5]. A variety of microorganisms such as bacteria, including Staphylococcus aureus and Streptococcus species, Mycobacterium tuberculosis, and Treponema pallidum have been implicated in the etiology. Rarely, fungi such as Candida and Aspergillus, and organisms like Actinomyces have been seen as causative organisms [6-8].

The pathophysiology of mycotic pulmonary aneurysm includes involvement of pulmonary artery from a focus of suppurating or cavitating pulmonary infection, as in tuberculosis, in which case it is termed as a Rassmusen aneurysm. Other causes include ischemic insult to the arterial wall due to infection of the vasa vasorum, as seen in syphilis. There may also be a direct extension into the arterial wall from an endoluminal septic thromboembolus seen in cases of bacterial endocarditis [2-3]. Virulent microorganisms cause extensive destruction of the layers of the arterial wall and usually result in the formation of a false aneurysm or pseudoaneurysm. On the other hand, indolent organisms often cause a true aneurysm, as in these instances the arterial wall is less severely destroyed [7].

The plain radiographic appearance of a mycotic pulmonary aneurysm is a pulmonary opacity which may be either well-defined or ill-defined. There may be parenchymal consolidation as well, which is generally non-specific and cannot be differentiated from infections and neoplasms. Although conventional angiography was previously considered the gold standard of diagnosis, cross-sectional imaging such as contrast-enhanced CT and MRI are now important alternative imaging modalities which clearly demarcate the vascular origin of the lesion from the pulmonary vessels [3,6-7]. In our patient, CT scan showed a hyper-enhancing lesion which was connected to a pulmonary artery on the arterial phase and showed surrounding area of low attenuation which was the thrombosed component. The density of the enhancing focus had the same attenuation as the enhancing vessels, which was therefore virtually diagnostic of a pulmonary aneurysm.

Behçet’s disease is a vasculitis of unknown etiology. Large-vessel involvement may be observed in 15% to 35% of patients and pulmonary artery aneurysms may develop in the course of the disease [3]. Therefore, Behçet’s disease was considered in the differential diagnosis of the reported case. However, according to the diagnostic criteria proposed by the International Study Group for Behçet’s disease, recurrent oral ulceration must be present as well as at least two of the following: recurrent genital ulceration, uveitis, skin lesions, or a positive pathergy test. Our patient had none of these and Behçet’s disease was easily excluded on the basis of clinical findings. Moreover, the differential diagnosis of a mycotic pulmonary artery aneurysm can be difficult to establish on imaging especially when there are no secondary signs such as pneumonic consolidation adjacent to the aneurysm and presence of cavitatory nodules which are suggestive of a septic thromboembolic source. These secondary findings were not seen in our patient and therefore we did not consider mycotic aneurysm as a differential initially until the echocardiogram was done.

Experience in the management of mycotic pulmonary aneurysms is limited as their diagnosis is rare and infrequently reported in the literature. Management is usually surgical and involves aneurysmectomy, lobectomy, aneurysmorrhaphy, or banding. Additionally, alternative endovascular techniques, such as occlusion of aneurysm with steel coils or detachable balloons have been reported [8-9]. However, conservative management is chosen when there is no evidence of acute hemoptysis or other emergent symptoms, or when a patient is not a candidate for intervention.

Spontaneous resolution has been previously reported for small mycotic pulmonary artery aneurysms but regular follow-up CT scans are mandatory [4]. Surgical treatment should always be undertaken to prevent catastrophic consequences like rupture for large aneurysms. Direct ligation of the feeding vessel and endoaneurysmorrhaphy with preservation of lung tissue has been advocated as the ideal surgical technique; however, it is rarely feasible and practicable as reported by Westaby et al [9]. The prognosis of mycotic pulmonary artery aneurysms is not well established; however, high mortality rates of more than 50% have been previously reported [2].

## Conclusions

This article highlights the manifestation and clinical behavior of multiple mycotic pulmonary artery aneurysms in a patient with infective endocarditis. A close follow-up with radiological modalities demonstrated progressive enlargement and rupture of the left-sided aneurysm which proved fatal for the patient before any treatment could be sought. This case also emphasizes that when pulmonary artery aneurysms are found on imaging without other overt signs of lung infection, it is important to consider a diagnosis of mycotic pulmonary aneurysm and proceed further with cardiac evaluation.
